# Heterozygosity is low where rare color variants in wild carnivores prevail

**DOI:** 10.1002/ece3.10881

**Published:** 2024-02-07

**Authors:** Laura Tensen, Klaus Fischer

**Affiliations:** ^1^ Zoology, Institute for Integrated Natural Sciences Koblenz University Koblenz Germany; ^2^ Department of Zoology, Centre for Ecological Genomics and Wildlife Conservation University of Johannesburg Johannesburg South Africa

**Keywords:** anomalous colouration, Carnivora, color morphs, genetic diversity, inbreeding, population bottleneck

## Abstract

Coat color and pattern are a distinguished feature in mammalian carnivores, shaped by climatic cycles and habitat type. It can be expressed in various ways, such as gradients, polymorphisms, and rare color variants. Although natural selection explains much of the phenotypic variation found in the wild, genetic drift and heterozygote deficiency, as prominent in small and fragmented populations, may also affect phenotypic variability through the fixation of recessive alleles. The aim of this study was to test whether rare color variants in the wild could relate to a deficiency of heterozygotes, resulting from habitat fragmentation and small population size. We present an overview of all rare color variants in the order Carnivora, and compiled demographic and genetic data of the populations where they did and did not occur, to test for significant correlations. We also tested how phylogeny and body weight influenced the presence of color variants with phylogenetic generalized linear mixed models (PGLMMs). We found 40 color‐variable species and 59 rare color variants. In 17 variable phenotypic populations for which genetic diversity was available, the average *A*_R_ was 4.18, *H*_O_ = 0.59, and *H*_E_ = 0.66, and *F*_IS_ = 0.086. We found that variable populations displayed a significant reduction in heterozygosity and allelic richness compared to non‐variable populations across species. We also found a significant negative correlation between population size and inbreeding coefficients. Therefore, it is possible that small effective size had phenotypic consequences on the extant populations. The high frequency of the rare color variants (averaging 20%) also implies that genetic drift is locally overruling natural selection in small effective populations. As such, rare color variants could be added to the list of phenotypic consequences of inbreeding in the wild.

## Graphical abstract

We present an overview of all rare color variants in the order Carnivora, and compiled demographic and genetic data of the populations where they did and did not occur, to test for significant correlations. We found that variable populations displayed a significant reduction in heterozygosity and allelic richness compared to non‐variable populations across species. It is likely that small effective size had phenotypic consequences on the extant populations, and that genetic drift is locally overruling natural selection.

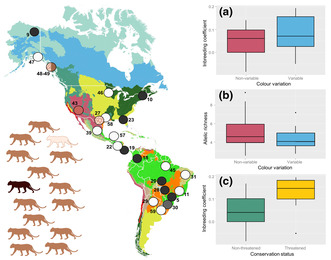

## 1 INTRODUCTION

**FIGURE 1:**
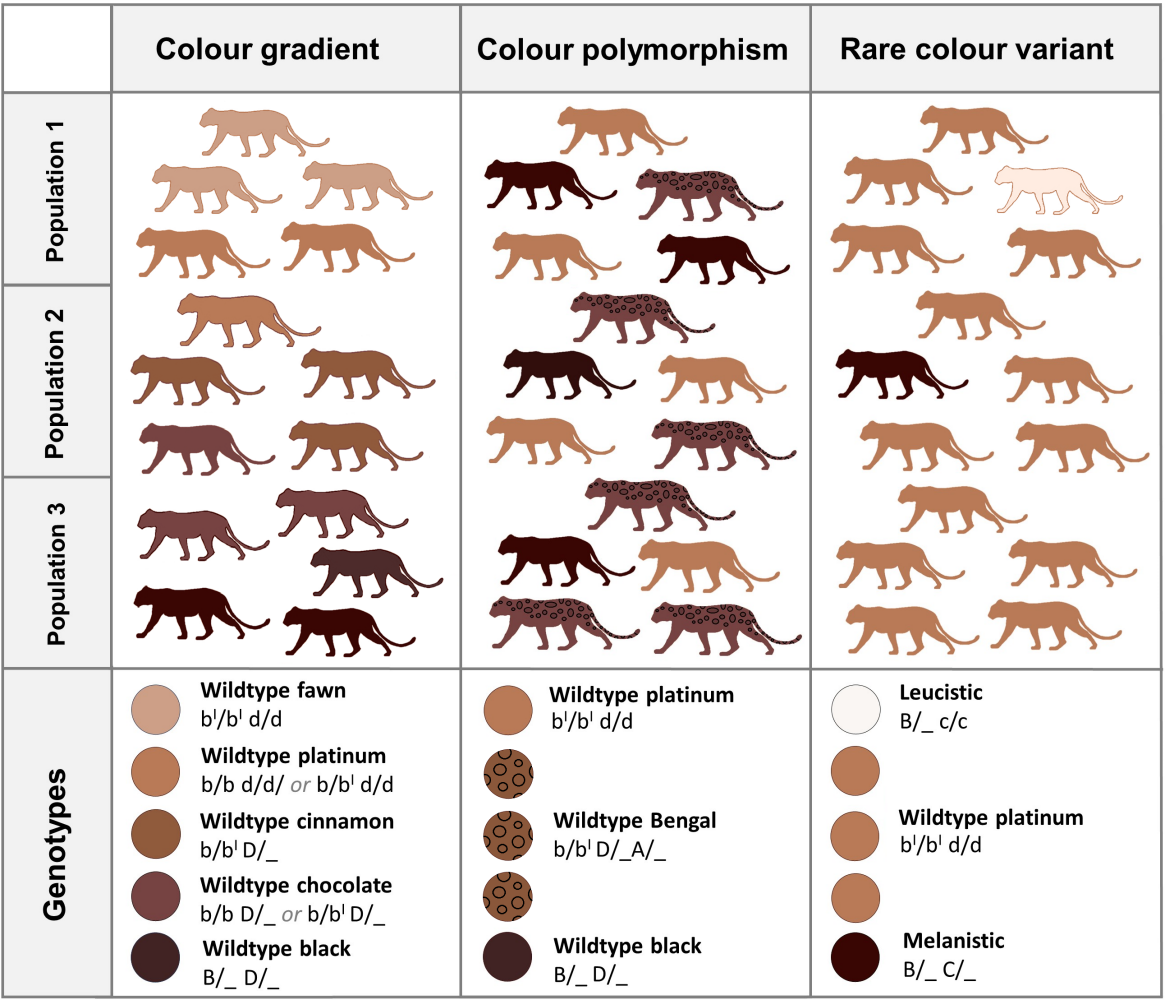
The difference between coat color gradients, color polymorphism, and rare color variants. As an illustrative example, genotypes consist of the B locus = Brown (these variants have reduced eumelanin and appear brownish in color); D locus = Dilution (causes uneven distribution of the pigment in the fur shaft); A locus = Bengal (the agouti signaling protein switches between black and red pigments, creating a banding pattern in individual hairs); C locus = Color restriction (through reduced or increased production of melanin). Rare color variants (in this example the C locus) can become fixed in populations (such as the D locus), after which it may be considered a new Wildtype color polymorphism.

Phenotypic variation occurs throughout the animal kingdom and has been widely used to investigate evolutionary processes such as balancing selection and sympatric speciation (McLean & Stuart‐Fox, 2014). Coat color and pattern, a type of phenotypic variation, have been of particular interest to evolutionary biologists due to their traceability in the wild (McKinnon & Pierotti, 2010). They are a prominent feature in mammalian carnivores, shaped by environmental factors such as past climatic cycles and habitat type (Eizirik et al., 2010). Coat color variation can be expressed in various ways, such as gradients, polymorphisms, and rare color variants (Figure 1). Color gradients occur in many species, in which intermediate phenotypes or clinal variation occur along latitudal gradients in response to changing vegetation types (McLean & Stuart‐Fox, 2014; Taylor et al., 1990). A famous example of ecogeographical habitat matching is the Gloger's rule, which describes how variation in coloration relates to climatic gradients, in which animals are more pigmented towards the equator (Caro, 2005; Delhey, 2019). It has been illustrated in, among many other taxa, hog‐nosed skunks *Conepatus leuconotus* (Ferguson et al., 2022) and striped skunks *Mephitis mephitis* (Walker et al., 2023), which have reduced whiteness towards Central America, related to canopy cover. Furthermore, reddish variants are more common in open dry habitats, whereas gray and black variants are more often associated with wet areas and tropical forests respectively (Da Silva et al., 2016, 2017). Complex coat patterns, such as spots or rosettes, are more frequently found in arboreal carnivores living in densely forested areas, whereas those with uniform coats more often occur in open habitats (Allen et al., 2011).

When clearly distinguished coat variations are established within a species range, albeit at different frequencies, it is often referred to as color polymorphism (Roulin, 2004). A species is considered polymorphic when individuals in a population display one or several color variants that are genetically inherited and which expression is unrelated to the environment, age, sex or body condition (Buckley, 1987; Roulin, 2004). Occasional rare color variants, which tend to be restricted to one population, are commonly not yet considered polymorphism at the species level. In the wild, rare color variants are normally lost through fixation unless they are maintained through a rare‐morph advantage or heterozygote advantage in stable populations (Gray & McKinnon, 2007). They may become abundant in populations if they have a selective benefit, such as for thermoregulation, sexual signaling, warning, or camouflage (Caro & Mallarino, 2020). Color polymorphism may also face disruptive selection in different microhabitats, due to which spatial or temporal niche segregation can be observed in the same area (Da Silva et al., 2017; Graipel et al., 2014; Nijhawan et al., 2019). As such, new traits may allow populations to occupy new niches and facilitate range expansion (Forsman et al., 2008). Color morphs could also be subjected to strong sexual selection, which can lead to assortative mating (Pryke & Griffith, 2007; Wellenreuther et al., 2014). Over time, this may lead to a speciation event, which implies that color polymorphic species may represent incomplete speciation (Wagner et al., 2012). In bird species, it was illustrated that color polymorphic species speciate more rapidly and give rise to monomorphic daughter species (Hugall & Stuart‐Fox, 2012).

Although natural selection, which may vary within a species' geographic range, explains much of the phenotypic variation found in the wild (Da Silva et al., 2016), genetic drift and heterozygote deficiency, as prominent in small populations, may also affect phenotypic variability through the fixation of recessive alleles (Eizirik et al., 2010). It has been suggested that recent changes in coat color or pattern may have resulted from population bottlenecks, caused by habitat fragmentation and human‐induced mortality (Kubala et al., 2020). Habitat fragmentation and human‐induced mortality reduce the effective size of animal populations and increases spatial isolation, which can lead to the erosion of genetic variation (Templeton et al., 1990). Specifically, inbreeding and genetic drift could result in reduced heterozygosity and allelic richness in isolated populations, which may increase the expression of rare alleles (Reed & Frankham, 2003). In other contexts, phenotypic change in small and isolated populations has already been confirmed, such as fluctuating asymmetry in brown bears *Ursus arctos* (Loy et al., 2021) and bone deformities in wolves *Canis lupus* (Räikkönen et al., 2009). It is therefore possible that the occurrence of rare color variants may also relate to small effective population sizes. Indeed, there is some evidence that genetic drift has increased the occurrence of rare color variants in wild carnivores, such as black tigers *Panthera tigris* (Sagar et al., 2021) and red leopards *P. pardus* (Tensen et al., 2022). At least in these cases, it is possible that small effective size had phenotypic consequences on the extant populations.

The prerequisite for color polymorphism is genetic variation through point mutations, or base pair deletions and duplications, which can evolve in allopatry or sympatry, and through hybridization (Roulin, 2004). As illustrated in the Figure 1, many different loci and genotypes can underlie color variants (Lyons, Foe, et al., 2005), and they can be inherited via dominant or recessive genes (Anderson et al., 2009). Important pathways involved with color variation are pigment‐type switching genes such as in the Melanocortin 1 receptor (MC1R) and Agouti signaling protein (ASIP) (Kaelin & Barsh, 2013). Typically, associated mutations are recessive and need to occur in homozygosity to affect the phenotype (Schneider et al., 2012). Due to recessive inheritance, increased frequencies of color variants in captive‐bred animals are normally achieved by establishing some level of inbreeding, increasing the level of homozygosity (Cieslak et al., 2011). For instance, many domestic cat breeds have originated from selecting missense point mutations with an autosomal recessive mode of inheritance in the tyrosinase (TYR) and tyrosinase‐related protein 1 (TYRP1) (Schmidt‐Küntzel et al., 2005), which is related to the albinism pathway (Lyons, Imes, et al., 2005). Therefore, rare color variants in the wild may also be associated with the expression of recessive alleles in small and fragmented populations.

The aim of our study was to test whether color variants in carnivores species could relate to a deficiency of heterozygotes in the wild. Although potential selective drivers cannot be ruled out, we consider that the occurrence and frequency of rare color variants are likely associated with the increased expression of recessive alleles in small and fragmented populations. Specifically, we test the following hypotheses: (i) populations in which rare color variants occur have a lower allelic richness and higher heterozygote deficiency than populations in which they are absent, (ii) rare color variants occur more frequently in areas that suffer from habitat fragmentation, and (iii) rare color variants occur more frequently in small populations. We tested these hypotheses on mammalian carnivores, because they are particularly rich in coat color and patterns (Eizirik & Trindade, 2021). Furthermore, carnivores are vulnerable to anthropogenic habitat loss, due to their large area and food requirements, and low population densities and reproductive rates, which particularly holds true for large carnivores (Ripple et al., 2014). We here present an overview of all rare color variants in the order Carnivora that have been reported in the wild, and compile data on genetic processes underlying these natural occurrences. By this means, we hope to gain more insights into the relationship between phenotypic variation in the wild and their underlying causes in small and fragmented populations.

## 2 METHODS

### 2.1 Literature review

We included all rare color variants that have been reported in scientific literature or online papers over the past five decades (from 1970 to 2023) and tried to retrieve genetic data from the population where the color variant occurred. We conducted common Google and scientific web searches (e.g., Google Scholar and Web of Science) to find rare color variants. We define a rare color variant as “a distinct phenotypic form of coat colour or pattern that typically occurs in a specific region and at low frequencies.”. To the contrary, color polymorphism is described as phenotypic variation that is well established throughout a species' range (Wildtype phenotypes), albeit at different frequencies. Hence, we do not consider a rare color variant to be polymorphism at the species level. Although rare color variants may become of adaptive significance in the future, counting them as polymorphism in this study may distort the correlation we make with genetic drift and homozygote access. We included all land‐living carnivores and only those species for which scientific publications with photographic evidence were available.

We used some of the following search strings: carnivores OR Carnivora OR family name (of all 12 Carnivora families) AND color morph OR anomalous coloration OR phenotypic variation OR leucistic OR melanistic. Scientific papers were also drawn from the reference list of the articles that were found. We did not list a color variant twice (i.e., from different locations), but instead included the best scientifically documented case for each species. We acknowledge that our approach is not holistic and prone to errors. For instance, camera traps can cause biases in human perception and are not always able to tell the difference between color variants such as pseudomelanism versus intraspecific coat variation or seasonal molting, or between leucism or albinism (Olson & Allen, 2019). Albinism is a recessively inherited disorder that occurs throughout all taxa, whereas leucism can have adaptive significance (Olson & Allen, 2019). Because albinism in a species does not mean the presence of color variation in a population, we have excluded this from our dataset. Overlooking or mislabelling color variants will most likely not affect our overall results, which aims to find a link between rare phenotypes and genetic diversity.

### 2.2 Parameters

We also searched the scientific literature to find a possible link between the color variation in populations and a possible cause of their frequency, such as a selective advantage, population bottleneck or heterozygote deficiency. Because the latter is merely speculative, we also tried to retrieve genetic data from the same population. In total, we noted for each color variant: the type (e.g., dilution, melanism, leucism), geographic location, speculated cause, gene mutation, frequency, allelic richness (*A*_R_), observed (*H*_O_) and expected (*H*_E_) heterozygosity, and inbreeding coefficient (*F*_IS_), when such data were available. All genetic papers were based on microsatellites, with a minimum of 5 loci. When *F*_IS_ values could be retrieved, we compared these to the body size, population size, and level of habitat fragmentation in the area where the color variable population occurs, to draw potential correlations. Because exact population sizes are often difficult to retrieve, they were divided into categories (<50, <250, <1000, >1000 adult individuals), derived from literature. The level of habitat fragmentation was divided into categories (high, medium, low) based on metrics provided by Crooks et al. (2017).

### 2.3 Statistical analysis

To calculate statistical correlations and differences between populations at the 5% level of significance, we performed Kruskal–Wallis H and Wilcoxon rank tests for multiple groups, and Mann–Whitney *U*‐tests for two groups, using R (R Core Team). We also compared numerical data of color variable populations to non‐variable conspecifics that are less constrained by population and/or habitat size. In our analyses, the dependent variable (genetic diversity) and independent variables (body size, population size, and level of habitat fragmentation) were compared. However, because the species included in our study are mainly found in three different taxonomic families, the data lacks independence, due to which we calculated the difference between variable and non‐variable populations for each metric for each species, assuming that what happens within each species in terms of population divergence is independent of what is happening in other species. From this difference data for each species, we calculated 95% Confidence Intervals (CI) with an independent *T*‐test to determine whether the mean difference was different from zero. This was done for allelic richness, expected heterozygosity, and inbreeding coefficients. All significant correlations were visualized with ggplot2 in R (Wickham et al., 2016).

We also tested how body weight influenced color variants in species with a phylogenetic generalized linear mixed models (PGLMMs), which estimates regression coefficients for binary and continuous data while incorporating interspecific relatedness (Ives & Helmus, 2011). A phylogeny and data frame of 70 carnivore species (Data S1) were adopted from Diniz‐Filho and Tôrres (2002), to which color variant presence/absence data were added (Data S2). We evaluated PGLMMs in ‘phyr’ 1.0.3 R package (Li et al., 2020), to test whether body weight (log‐transformed) was associated with the presence of color variants. Log transformation normalizes the strong right skew in metrics, which is recommended for comparative approaches (Ives & Garland, 2014). Because species only occur once as tips of the phylogeny, we used the pglmm_compare() function. Range size (log‐transformed), and phylogenetic covariance were set as a random factor in our models, and results were presented with standard error (SE). We calculated *R* ^*2*^ for PGLMM models using the package ‘rr2’ 1.0.2 (Ives et al., 2019).

## 3 RESULTS

**FIGURE 2:**
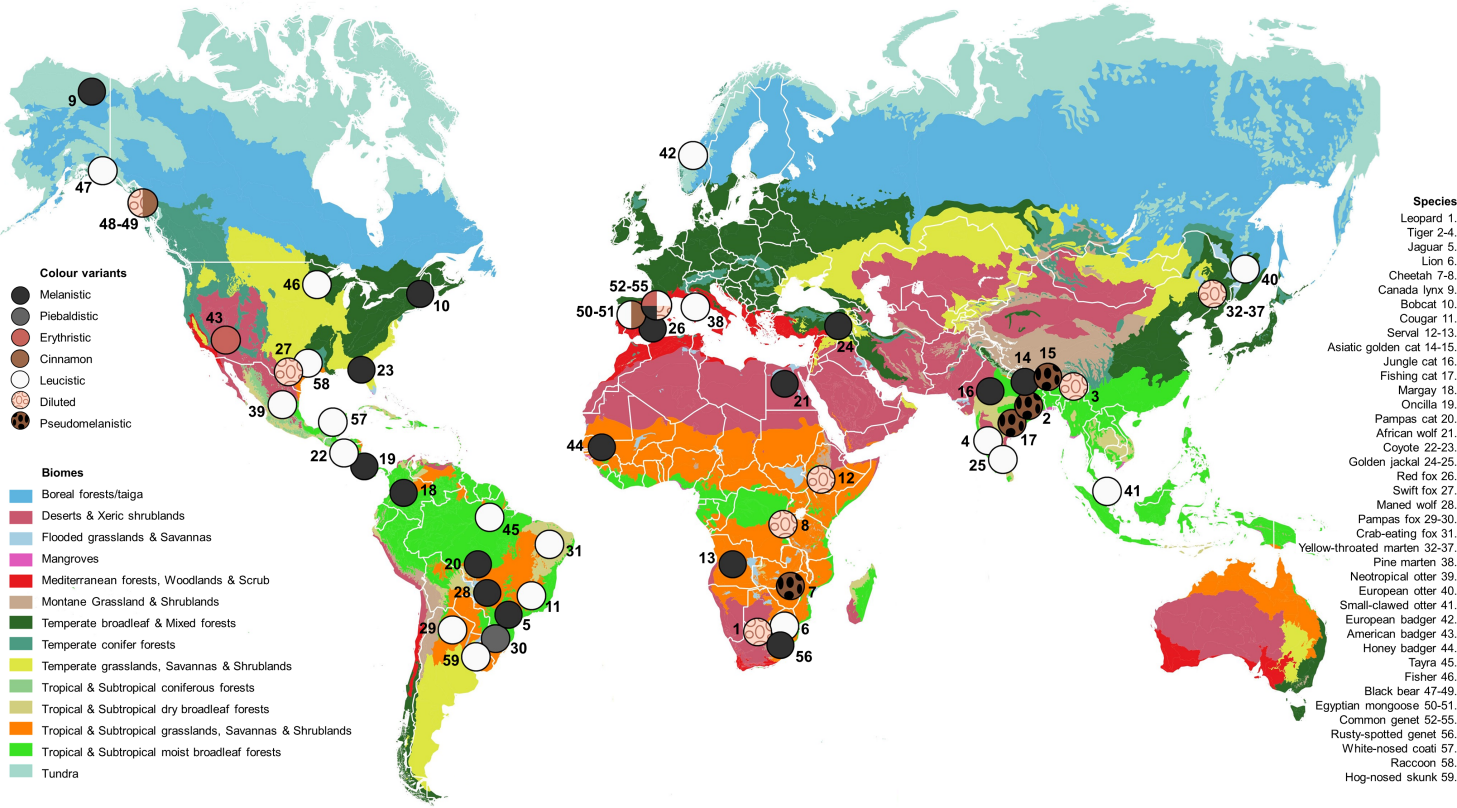
A map with all color morphs found in carnivores (*N* = 59). The entire list of color morphs and scientific names is found in Table S1.

We found a total of 40 color‐variable species and 59 rare color variants (Figure 2). Rare color variants were primarily found in the Felidae (*N* = 20), Mustelidae (*N* = 15) and Canidae (*N* = 11) families. Hyaenidae was the only carnivore family for which no color variants was detected. Twenty of the color variants were described as leucistic (34%), and 17 as melanistic (29%). The average frequency of color variants was 20% in the population where it occured, based on data from 16 publications.

### 3.1 Genetic parameters

**FIGURE 3:**
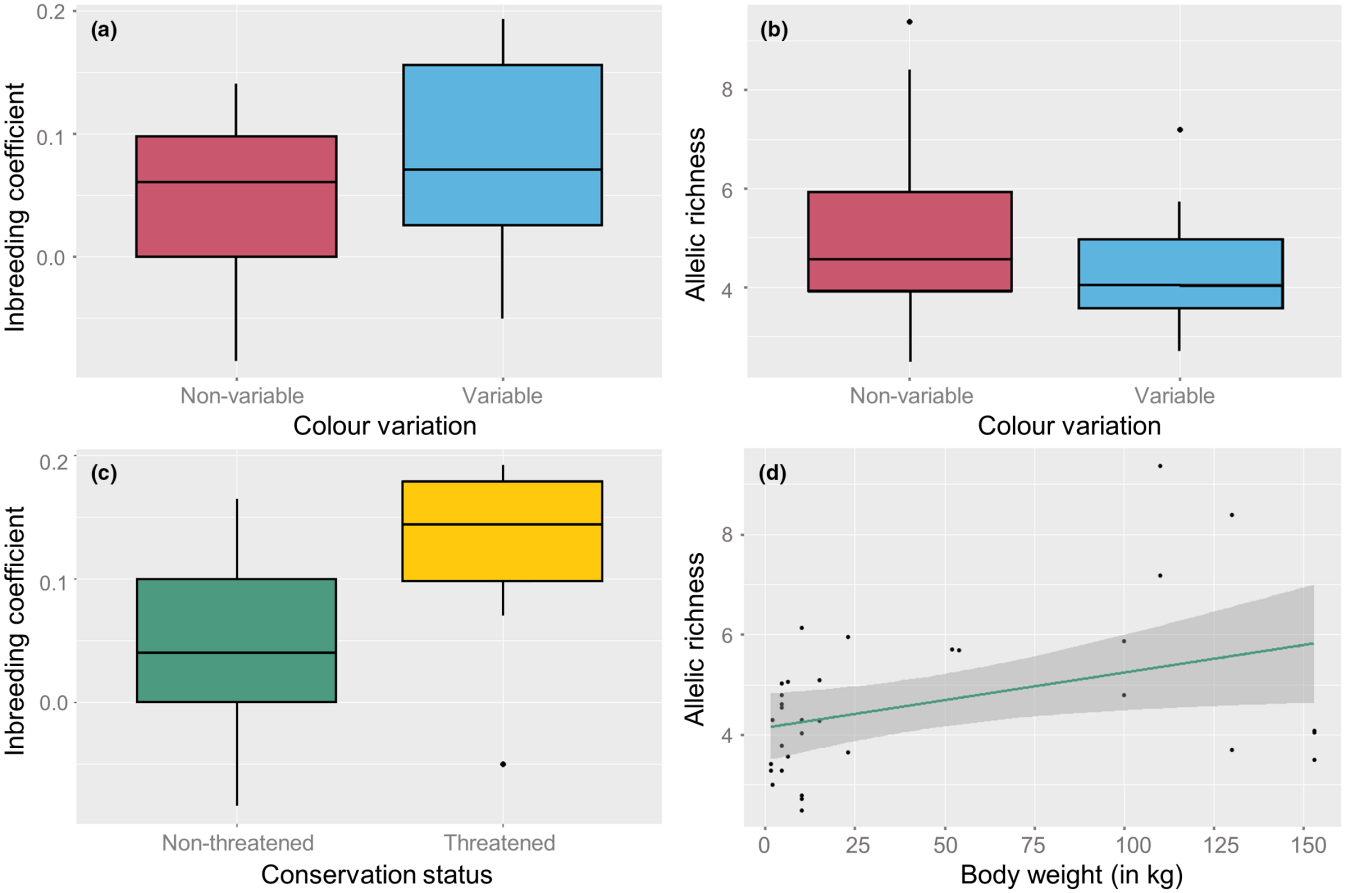
Correlations of genetic diversity and the presence of rare color variants. (a.) The relationship between inbreeding coefficient and variable phenotypic populations (*T*‐test, *t* value = 1.88; *p* = .034); (b) The relationship between allelic richness and variable phenotypic populations (*T*‐test, *t* value = −2.23; *p* = .016); (c) The relationship between inbreeding coefficients and populations with threatened (<250 individuals) and non‐threatened (>250 individuals) conservation status (Mann–Whitney; *W* = 194; *p* = .01); (d) The relationship between allelic richness and body weight in kilograms (kg) (Spearman; *R* = .36; *p* = .04).

Twenty publications speculated about the underlying cause of the color variant frequency, of which 10 papers hinted towards heterozygote deficiency (e.g., due to bottleneck, inbreeding, genetic drift), while 8 papers presumed a selective benefit. Three other papers speculated hybridization to be the underlying cause. In 17 variable phenotypic populations, there was data on genetic diversity available from the population or region containing the color variant, regardless of their frequency. In these populations, the average *A*_R_ was 4.18, *H*_O_ = 0.59, and *H*_E_ = 0.66 (Table 1). This means that we found an overall heterozygote deficiency, with an average *F*_IS_ of 0.086 (−0.05–0.19) across all color variable carnivores (the full dataset is available in Table S1). In non‐variable phenotypic populations, the average *A*_R_ was 5.09, *H*_O_ = 0.63, *H*_E_ = 0.66, and *F*_IS_ = 0.046 in non‐variable populations (Table S2). In large and stable leopard populations, the number of alleles normally ranges between 5 and 20 per locus, and heterozygosity levels are usually high (0.70–0.75) with no or little heterozygote deficiency (0.0–0.05) (Spong et al., 2000), which can serve as a proxy for other carnivore species. When comparing the pairwise increase of heterozygote deficiency from non‐variable populations to variable populations within species, we found a significant directional deviation from zero (*T*‐test, *t* value = 1.88; *p* = .034). This implies an increase of heterozygote deficiency in populations where rare color variants occurred (Figure 3a). We also found a negative deviation from zero for allelic richness (*T*‐test, *t* value = −2.23; *p* = .016), implying that variable populations have reduced genetic diversity Figure 3b.

**TABLE 1:** Demographic and genetic parameters of color variable (VAR) and non‐variable (NON) carnivore populations.

Species^a^	Demographic parameters			Genetic parameters			
	PS	Region	HF	*A*_R_	*H*_O_	*H*_E_	*F*_IS_
Leopard^VAR^	2	North West, South Africa	2	5.7	0.54	0.66	0.18
Leopard^NON^	4	Selous, Tanzania	3	–	0.77	0.84	0.06
Tiger^VAR^	2	Assam, India	1	4.05	0.71	0.82	0.14
Tiger^VAR^	3	Western Ghats, India	1	4.09	0.70	0.83	0.16
Tiger^NON^	2	Terai Arc, Nepal	1	3.51	0.54	0.61	0.11
Jaguar^VAR^	1	São Paulo, Brazil	1	7.19	0.68	0.73	0.07
Jaguar^NON^	4	Amazon, Brazil	3	9.38	0.67	0.76	0.11
Lion^VAR^	2	Reserves South Africa	2	4.79	0.55	0.71	0.18
Lion^NON^	4	Okavango, Botswana	3	5.88	0.65	0.69	0.06
Bobcat^VAR^	4	New Brunswick, Canada	3	4.04	0.66	0.68	0.03
Bobcat^NON^	4	Central United States	2	6.14	0.75	0.78	0.04
Cougar^VAR^	1	Rio de Janeiro, Brazil	1	5.71	0.56	0.78	0.17
Cougar^NON^	4	Wyoming, United States	3	–	0.54	0.54	0
Fishing cat^VAR^	3	Andhra Pradesh, India	1	2.80	0.46	0.45	−0.01
Fishing cat^NON^	2	Howrah, India	1	2.50	0.46	0.51	0.10
Coyote^VAR^	4	Florida, United States	1	5.09	0.86	0.85	−0.01
Coyote^NON^	4	Ohio, United States	3	4.29	0.86	0.85	−0.01
Golden jackal^VAR^	4	Artvin, Turkey	2	3.57	0.52	0.61	0.15
Golden jackal^NON^	4	Central Europe	2	5.07	0.47	0.51	0.08
Maned wolf^VAR^	2	Minais Gerais, Brazil	1	3.65	0.75	0.72	−0.05
Maned wolf^NON^	3	Central West Brazil	2	5.95	0.71	0.65	−0.07
Pine marten^VAR^	3	Elba Island, Italy	1	3.29	0.42	0.52	0.04
Pine marten^NON^	4	Ardennes, France	2	3.42	0.59	0.61	0.04
Small‐clawed otter^VAR^	2	Sumatra, Indonesia	1	4.62	0.59	0.73	0.19
Small‐clawed otter^NON^	3	Singapore	1	3.29	0.51	0.47	−0.09
European badger^VAR^	3	Orkdal, Norway	3	2.73	0.46	0.48	0.04
European badger^NON^	4	Scarborough, UK	1	4.30	0.52	0.61	0.14
Fisher^VAR^	4	Wisconsin, USA	2	3.79	0.48	0.49	0.02
Fisher^NON^	4	Maine, USA	3	4.55	0.60	0.61	−0.01
Black bear^VAR^	3	Gribbell, Canada	3	–	0.54	0.55	0.04
Black bear^VAR^	4	Yakutat, Alaska	3	3.71	0.59	0.58	0.02
Black bear^NON^	4	Minnesota, USA	3	8.40	0.79	0.79	0
Common genet^VAR^	4	Catalonia, Spain	2	3.00	0.47	0.52	0.13
Common genet^NON^	4	Algeria and Tunisia	2	4.30	0.62	0.63	0.09
White‐nosed coati^VAR^	3	Quintana Roo, Mexico	2	5.03	0.67	0.77	0.13
White‐nosed coati^NON^	4	Central Mexico	2	4.80	0.65	0.75	0.13

*Note*: Population sizes (PS) were divided over four categories: 1 = <50 adult individuals, 2 = <250 individuals, 3 = <1000 individuals, and 4 = >1000 individuals. The level of habitat fragmentation (HF) was divided in three categories: 1 = high, 2 = medium, 3 = low. Demographic and genetic parameters are retrieved from various sources, which are listed in Table S2. *A*_R_, allelic richness; *H*_O_, observed heterozygosity; *H*_E_, expected heterozygosity; *F*_IS_, inbreeding coefficient.

^a^ The following species are included: leopard *Panthera pardus*, tiger *P. tigris*, jaguar, *P. onca*, lion *P. leo*, bobcat *Lynx rufus*, cougar *Puma concolor*, fishing cat *Prionailurus viverrinus*, coyote *Canis latrans*, golden jackal *C. aureus*, maned wolf *Chrysocyon brachyurus*, pine marten *Martes martes*, small‐clawed otter *Aonyx cinereus*, European badger *Meles meles*, fisher *Pekania pennanti*, black bear *Ursus americanus*, common genet *Genetta genetta*, and white‐nosed coati *Nasua narica*.

### 3.2 Demographic parameters

In an attempt to correlate heterozygote deficiency to demographic data, we have retrieved the population size and level of habitat fragmentation of color variable populations (Table 1). The values of 17 variable populations were statistically compared to similar data of non‐variable populations that were less constrained by population or habitat size (an overview of all tests and results are available in Table S3). Low population size generally yielded higher *F*_IS_ values, but this relation was non‐significant (Kruskal–Wallis; *X* ^2^ = 7.25; *p* = .06). The correlation was tested significant when comparing threatened populations (<250 individuals) to non‐threatened populations (>250 individuals), in which threatened populations had higher inbreeding coefficients (Mann–Whitney; *W* = 194; *p* = .01; Figure 3c). Although we found a weak positive correlation, a higher level of habitat fragmentation did not yield significantly higher inbreeding coefficients (Kruskal–Wallis; *X* ^2^ = 4.96; *p* = .08). We did find that body weight negatively correlates with allelic richness (Spearman; *R* = .36; *p* = .04; Figure 3d) and expected heterozygosity (Spearman; *R* = .40; *p* = .02). To account for phylogenetic nonindependence of our species data due to shared ancestry, we used a PGLMM to test the effect of body weight on the presence of rare color variants. The results suggests that variants do not vary in their occurrence in relation to body weight (PGLMM coefficient: 0.069 ± 0.088 SE, *Z* = 0.579, *p* = .42). The intercepts for species were tested significant, meaning that close relatives had similar values (PGLMM coefficient: 0.93 ± 0.45 SE, *Z* = 2.04, *p* = .04). Thus, the results imply an overall phylogenetic effect, and no (or little) body weight effect. The *R* ^2^ value suggests that body weight alone explains approximately 15.9% of color variation in populations, whereas this is reduced to 11.5% if phylogeny is incorporated.

## 4 DISCUSSION

The aim of our study was to test whether rare color variants in the wild may relate to a deficiency of heterozygotes, possibly resulting from habitat fragmentation and reduced effective population size. We found that variable populations displayed a significant reduction in heterozygosity and allelic richness compared to non‐variable populations across species. We also found a significant correlation between population size and inbreeding coefficients, in which small populations suffer from larger heterozygote deficiencies. This supports the hypothesis that heterozygote deficiency in small populations might have an influence on the prevalence of rare color variants due to the expression of rare recessive alleles. The high frequency of the rare color variants in some populations also implies that genetic drift is occurring in small populations.

In 17 variable populations for which we found genetic data, there was some indication of a heterozygosity deficiency. This was particularly evident in charismatic species such as black tigers (Kolipakam et al., 2019), red leopards (Tensen et al., 2022), and white cougars *Puma concolor* (Saranholi et al., 2017). Non‐variable populations of many species also suffered from heterozygote deficiencies due to demographic losses, but the difference with non‐variable populations was nonetheless profound in some cases and significant across species. The direct comparison between variable and non‐variable populations remains challenging, because stochastic events or loss of rare variants will interact with selective pressures (Caro & Mallarino, 2020). Furthermore, as our data are based on literature, we were not always able to include the populations of choice or retrieve historic frequency and presence/absence data. Studies with different sample sizes were also compared, which introduces the risk of bias, and the PGLMM showed an influence of phylogeny on color variant presence. Despite these limitations, the loss of genetic diversity in populations where rare color variants prevail is remarkable, and adds to the body of knowledge on the phenotypic effects of inbreeding in wild populations. The level of habitat fragmentation did not appear to influence the presence of rare color variants, possibly because our dataset did not allow for enough fine‐scale detail. The relationship between habitat fragmentation and genetic diversity remains indisputable (Keyghobadi, 2007; Lino et al., 2019; Templeton et al., 1990), and has been linked to rare color variants before, such as for Glacier and Spirit bears *Ursus americanus* in Alaska (Lewis et al., 2020), where fjords act as barriers to gene flow. Likewise, spatial variation appears to be the primary driver of color morph variation in dragon lizard *Ctenophorus decressi* in Australia (McLean et al., 2015).

Another effect of geographic dispersal barriers is that local effective population sizes are smaller, increasing the effect of genetic drift in relation to migration and selection as an evolutionary force (Runemark et al., 2010). A striking example of genetic drift in small populations is the tiger population in the isolated Similipal tiger reserve, India, where pseudomelanism reached a frequency of 78%, and in parallel an exceptionally low heterozygosity was observed (*H*_O_ = 0.28) (Sagar et al., 2021). It has been revealed that nearly 14% of homologous alleles, both neutral and damaging, were fixed in small and isolated tiger populations (Khan et al., 2021). India has a particularly high degree of habitat fragmentation, and 56 mammalian color variants have been recorded between 1886 and 2017 (Mahabal et al., 2019). A similar situation appears to occur in the highly fragmented Atlantic rainforest, where melanism in jaguars *Panthera onca* is increasing, reaching frequencies of up to 38% (Haag, Santos, Sana, et al., 2010; Haag, Santos, Valdez, et al., 2010). A leucistic cougar has been spotted in the same area, which was speculated to be a sign of inbreeding (Cronemberger et al., 2018). Likewise, in Eurasian lynx *Lynx lynx* a wide range of natural coat patterns exists, associated with geographic location (Darul et al., 2022), which frequencies have recently altered due to population bottlenecks resulting from habitat fragmentation (Kubala et al., 2020). In South Africa, red leopards are spreading in northern provinces, where effective population sizes are low due to high local population offtake (Tensen et al., 2022). Even though this population is not isolated, high population turnover has led to natal philopatry, increasing the overall relatedness in the population and chances of recessive alleles being expressed.

We expect this trend to hold true for many other wild carnivores, in which genetic drift is locally overruling natural selection in small effective populations. For instance, phenotypic differences in Arctic foxes *Vulpes lagopus* reflect distinct selective advantages across their range; however, a recent increase of blue homozygotes in Arctic foxes *Vulpes lagopus* Scandinavia is considered the result of genetic drift (Tietgen et al., 2021). Furthermore, it appears that melanistic and leucistic genets *Genetta genetta* and mongoose *Herpestes ichneumon* are much more common in Europe, where they have been introduced, compared to their natural range in Africa (Delibes et al., 2013; Descalzo et al., 2021). Although it has been suggested that the phenotypic variation in Spain may relate to a relaxation of selective pressures (Descalzo et al., 2021), the founder effect has been overlooked. Likewise, melanistic wolves are common in Yellowstone National Park, United States, where they were introduced from in and around Jasper National Park, Canada, where melanism is also common (Hedrick et al., 2014). Melanism has reached frequencies of approximately 50% in these areas, which is unlikely to relate to a concealment advantage (Dekker, 2009) or reproductive fitness (Stahler et al., 2013), and may instead be the result of genetic drift. The binary appearance of melanistic wolves, as well as coyote, across their range (Hinton et al., 2022) supports the notion that neutral, rather than selective, processes are at play. In European badgers *Meles meles*, a population has recently established in central Norway where leucism now seems abundant, likely as a result of a founder effect (Hofmeester et al., 2021). The same could be the case for a leucistic coyote *Canis latrans* in Costa Rica (Arroyo‐Arce et al., 2019), and melanistic bobcats at the periphery of their range (McAlpine, 2021), although this is speculative.

A general trend of an increase in melanistic morphs towards the tropics and leucistic morphs towards the poles, as predicted by the Gloger's rule (Caro & Mallarino, 2020), could not be detected in this study. For instance, melanistic bobcats *Lynx rufus* are readily found in the northern tip of the United States (McAlpine, 2021) and Florida (Regan & Maehr, 1990), and a melanistic Canada lynx was found in the northern tip of Alaska (Jung, 2023). Although the Canada lynx appears to be a single sighting and is likely to suffer a selective disadvantage due to increased visibility, melanistic bobcats have become fairly common (McAlpine, 2021). When looking at the map of color morphs, we can also see the presence of many leucistic animals in the tropical forests of Mid‐America, such as leucistic coyote *Canis latrans*, coati *Nasua narica*, and neotropical otters (*Lontra longicaudis*). Furthermore, leucistic tayra *Eira barbara* have become abundant in the Guyana shield in Brazil (Mendes Pontes et al., 2020). This may further strengthen the theory that rare color variants are a random, sometimes maladaptive, occurrence, instead of a selective driver in response to spatially or temporally variable environments (Svensson, 2017). When not influenced by genetic drift, rare color variants can only be maintained through balancing or frequency‐dependent selection (Gray & McKinnon, 2007), which seems unlikely for the before‐mentioned examples. Furthermore, color variants will only be fixed throughout the range of a species when they have a selective benefit, at which stage they are normally referred to as color polymorphism (Svensson, 2017). Ecogeographical rules, as such, are more likely to imply to the latter category, and not to rare color variants that seems more selectively neutral or disadvantageous.

It has previously been suggested that leucism mainly prevails in the Mustelidae, based on the number of published studies (Olson & Allen, 2019), for which we found no evidence during this study. In general, it appeared that color variants are most abundant in the Felidae family (37.5% of cat species), representing 20 out of 59 color variants recorded in this study. To compare, the proportion of species that contain rare color variants is 23% in Canidae, 15% in Mustelidae, 12.5% in Ursidae, and 3% in Herpestidae and Viverridae (Table S1). Therefore, the high frequency of variable cats can be considered somewhat unusual. Furthermore, the PGLMM has illustrated a strong effect of phylogeny on the presence of color variants, implying shared inherited traits among closely related taxa. Because phenotypic variation is a driver of genetic variation, it is possible that Felidae species contain more protein polymorphisms associated with pigment‐type switching genes (Andersson & Purugganan, 2022). A similar range of coat patterns is observed in domestic cats, suggesting it is a conserved mechanism that can be altered by selection (Kaelin et al., 2012). Wild cat species exhibit the greatest diversity in colors and spots of all terrestrial carnivores, likely because they are more reliant on camouflage compared to for instance canids, which rely more on the chase (Darul et al., 2022; Schneider et al., 2012). However, it is worth noting that even though polymorphisms in wild canids are rare, domestic dogs show more phenotypic variation than any other domesticated animal (Kaelin & Barsh, 2013). This is due to genome‐wide slippage mutations and pure repetitive sequences in domestic dogs, which occur at a much greater rate than in other carnivores, likely resulting from strong population bottlenecks associated with the origin of dog‐breeds (Laidlaw et al., 2007).

Although we found that body size is generally a poor predictor of color variation in populations, we did find a correlation between allelic richness and body weight. Furthermore, a high proportion (33%) of the color variants occurred in large carnivore species (>10 kg), even though they have a low species diversity (representing 18% of all carnivore species). For instance, six of the listed color variants were found in large cats (*Panthera* spp.), of which only five species exist. The potential bias towards large carnivores could also partially result from the fact that they are more extensively studied and more easily observed (Tensen, 2018). They also suffer more from demographic and genetic deterioration compared to medium‐sized and small predators, based on relatively low effective population sizes (Lino et al., 2019). Even though we found no significant trend that apex predators contain more color variants, this was previously found in birds. While polymorphisms occur in approximately 3.5% of all bird species, an estimated 30% of raptorial species are known to be polymorphic (Hugall & Stuart‐Fox, 2012). In birds, color polymorphism was associated with accelerated speciation in avian apex predators, due to a larger niche breath, consistent with theoretical models where speciation is driven by the fixation of one or more morphs (Hugall & Stuart‐Fox, 2012). Polymorphic bird species also tended to be younger than monomorphic species, with shorter phylogenetic branches, which can further increase speciation rates (Forsman et al., 2008; Gray & McKinnon, 2007). For future research, it would be interesting to test whether this holds true for polymorphic carnivores as well.

In conclusion, we have collected some compelling evidence that heterozygote deficiency might contribute to the prevalence of rare color variants in the wild, due to the expression of recessive alleles following reduction in effective population size. As such, it can be added to the list of phenotypic consequences of population bottlenecks in the wild, alongside fluctuating asymmetry and bone deformities. Further investigation of phenotypically variable populations is needed to determine the genetic basis and the adaptive and evolutionary significance of rare color variants to help preserve genetic diversity. Ideally, a more comprehensive future study should directly sample, genotype, and compare variable and non‐variable populations of species. With an increase in genomic studies, we may also gain more insights into random processes versus directional selection in small and fragmented populations, and their relationship to color variation in the wild.

## AUTHOR CONTRIBUTIONS

**Laura Tensen:** Conceptualization (equal); data curation (equal); formal analysis (equal); investigation (equal); methodology (equal); writing – original draft (equal). **Klaus Fischer:** Supervision (equal); writing – review and editing (equal).

## CONFLICT OF INTEREST STATEMENT

The authors declare no competing interests.

## Supporting information


Appendix S1

File S1

File S2

## Data Availability

All data generated or analyzed during this study are included in this published article and its Supplementary Information files.
